# Treatment of Acute Kidney Injury Using a Dual Enzyme Embedded Zeolitic Imidazolate Frameworks Cascade That Catalyzes *In Vivo* Reactive Oxygen Species Scavenging

**DOI:** 10.3389/fbioe.2021.800428

**Published:** 2022-01-04

**Authors:** Xinyue Hou, Jianxiang Shi, Jie Zhang, Zhigang Wang, Sen Zhang, Ruifeng Li, Wei Jiang, Tingting Huang, Jiancheng Guo, Wenjun Shang

**Affiliations:** ^1^ Department of Kidney Transplantation, The First Affiliated Hospital of Zhengzhou University, Academy of Medical Sciences, Zhengzhou University, Zhengzhou, China; ^2^ Department of Molecular Pathology, Application Center for Precision Medicine, The Second Affiliated Hospital of Zhengzhou University, Academy of Medical Sciences, Zhengzhou University, Zhengzhou, China; ^3^ College of Chemistry, Jilin University, Changchun, China

**Keywords:** metal-organic frameworks, biomimetic mineralization, dual-enzyme cascade, ROS scavenging, acute kidney injury

## Abstract

Significant advances have been made in recent years for the utilization of natural enzymes with antioxidant properties to treat acute kidney injury (AKI). However, these enzymes have been of limited clinical utility because of their limited cellular uptake, poor pharmacokinetic properties, and suboptimal stability. We employed a novel biomimetic mineralization approach to encapsulate catalase (CAT) and superoxide dismutase (SOD) in a zeolitic imidazolate framework-8 (ZIF-8). Next, this SOD@CAT@ZIF-8 complex was anchored with MPEG_2000_-COOH to yield an MPEG_2000_-SOD@CAT@ZIF-8 (PSCZ) composite. The composite was then used as a stable tool with antioxidant properties for the integrated cascade-based treatment of AKI, remarkably improved intracellular enzyme delivery. This dual-enzyme-embedded metal-organic framework could effectively scavenge reactive oxygen species. In conclusion, the ZIF-8-based “armor plating” represents an effective means of shielding enzymes with improved therapeutic utility to guide the precision medicine-based treatment of AKI.

## 1 Background

Acute kidney injury (AKI) is a severe condition wherein glomerular filtration is impaired, leading to the accumulation of nitrogenous waste in the systemic circulation (Bellomo et al., 2012). Over time, AKI can progress to end-stage renal disease (ESRD), which affects up to 20% of hospitalized patients and results in high morbidity and mortality rates (Susantitaphong et al., 2013). Clinicians often encounter two forms of AKI, such as rhabdomyolysis-induced AKI (RM-AKI) or cisplatin-induced AKI (CP- AKI), although other instances exist (Arany and Safirstein, 2003). RM- AKI often occurs after the breakdown and necrosis of skeletal muscle releases pernicious proteins, largely myoglobin, and electrolytes into circulation. CP-AKI frequently arises from the nephrotoxicity associated with the use of cisplatin in the cancer patients, which occurs in about 1/3 of all patients (Pabla and Dong, 2008). Oxidative stress leading to acute tubular necrosis from reactive oxygen species (ROS) has been implicated in the pathogenesis of both conditions, as in many cases of AKI.

Reactive oxygen species (ROS), such as singlet oxygen (^1^O_2_), superoxide anions (O_2_), hydroxyl radicals (^•^OH), and hydrogen peroxide (H_2_O_2_), are the metabolites of normal cells in living organisms (Yasui and Sakurai, 2000; Ighodaro and Akinloye, 2018). Healthy kidney tissues can tolerate limited ROS levels with no adverse effects. However, excessive ROS can trigger a series of destructive processes in normal cells, including cell death and inflammation (Nath and Norby, 2000; Molitoris et al., 2007). Patients with AKI have excessive oxidative stress due to increased oxide activity and decreased antioxidant activity in their serum, leading to renal dysfunction. Unfortunately, no effective clinical treatments for AKI have been developed to date, and most of the patients have to seek supportive treatment via hemodialysis, peritoneal dialysis, and kidney transplantation. So how to prevent and treat AKI plays a pivotal role in clinical settings (Paller et al., 1984; Sureshbabu et al., 2015; Du et al., 2017).

Eliminating excess ROS can protect tissues from oxidative stress, and effectively scavenging excess ROS can, therefore, have a significant therapeutic effect on AKI (Dennis and Witting, 2017).

A range of antioxidant enzymes, including superoxide dismutase (SOD), catalase (CAT), and reduced glutathione (GSH), can function as ROS scavengers to mitigate oxidative stress within a given cellular environment (Savelieff et al., 2019; Wu et al., 2019). Given that they are enzymes rather than small molecule therapeutics, these enzymes can catalyze the sustained conversion of toxic ROS species into less damaging byproducts, with SOD facilitating the conversion of ^•^O_2_^−^ into H_2_O and then CAT converting H_2_O_2_ into water and O_2_ (Ferreira et al., 2018). While they can function in concert, CAT is primarily within peroxisomes, whereas SOD is typically in the cytosol or mitochondria, so additional transportation is necessary to facilitate H_2_O_2_ degradation (Sun et al., 2018; Wang et al., 2019). These endogenous enzyme systems show promise for *in vivo* therapeutic ROS scavenging and associated AKI treatment as adequate and well-controlled antioxidants. Such enzymatic cascade reactions have advantages over conventional multi-stage reactions, reducing barriers to diffusion while increasing local intermediate concentrations to enhance overall reaction efficiency at a molecular level. These good therapeutic properties have led many research groups to design enzymatic scaffold systems to mimic these efficient cascade systems in clinical settings. The *in vivo* application of these enzyme-based platforms would represent a key breakthrough with the potential to improve patient outcomes. However, several significant barriers currently limit the *in vivo* deployment of these enzyme-based systems, including the fact that they can readily undergo enzymatic degradation, are prone to enzyme aggregate formation, lose catalytic activity throughout prolonged storage, and exhibit poor uptake by target cells owing to their negatively charged surfaces. Embedding or encapsulating these enzymes in a solid carrier framework thus offers an opportunity to overcome these delivery and stability limitations.

Metal-organic frameworks (MOFs) are novel biocompatible materials that exhibit tunable porosity and a high surface area, making them ideal for the packaging of candidate therapeutic biomolecules (Simon-Yarza et al., 2017; Lu et al., 2018; Suresh and Matzger, 2019; Rosenkrans et al., 2020). These merits endow MOFs with considerable potential to host a wide variety of guests from gas molecules, biomicromolecules to nanoparticles for advanced applications. For example, enzymes are a kind of bionanopaticles and hosting enzymes using MOFs is an emerging immobilization biotechnology that circumvents the low loading efficiency, incompact confinement, and restricted pore size of the supports drawbacks caused by conventional porous solids. Traditionally, four primary strategies have been used for such packaging: grafting, infiltration, physical absorption, and encapsulation (Feng et al., 2015; Shieh et al., 2015; Jiang et al., 2017; Chen et al., 2021). However, these approaches do not allow for precise control of the size of the generated biomacromolecular-MOF composites and are often highly inefficient. In the past few decades, anchoring enzymes to the outer surface of MOF particles by chemical grafting or physical adsorption has been the primary method for entrapment of the enzymes (Rosenkrans et al., 2020; Tripathy et al., 2021). It relied on the interaction between the organic ligand of MOFs and the surface components of enzymes (Liu et al., 2021; Velásquez-Hernández et al., 2021; Xu et al., 2021). Although this method provides a certain protection against enzyme denaturation, most enzymes are still directly exposed, so immobilized enzymes are still susceptible to changes in the external environment (Liang et al., 2021; Velásquez-Hernández et al., 2021). Using the biomimetic mineralization strategy, encapsulating the enzyme within MOFs cavities (enzymes@MOFs) offered a aolution to the above problems (Liang et al., 2015; Rosenkrans et al., 2020). Leveraging the fact that biomacromolecule exhibit an affinity for metal ions to conduct rapid macromolecule embedding a MOF through the stimulation of zeolitic imidazolate framework-8 (ZIF-8) growth. This approach can shield the enclosed macromolecules within a porous ZIF-8 exoskeleton capable of protecting against denaturation while permitting selective transportation through this porous network structure. ZIF-8 exoskeletal constructs are highly stable and biocompatible under physiological conditions, while the entrapped enzymes were feasible to be released through simple modification of pH owing to the acid-sensitive nature of ZIF-8. The occluded enzymes could well maintain their activity. Most studies of such MOF bio-composites to date have explored their use as biosensors or catalytic agents in aqueous or organic solutions (Sun et al., 2012; Wang et al., 2017; Huang et al., 2020). Using these MOFs to deliver proteins or nucleic acids is, in contrast, a relatively novel approach (Zhuang et al., 2020a; Zhuang et al., 2020b). To our knowledge, no prior studies described the biomimetic mineralization-based encapsulation of dual-enzyme within MOF crystals to enhance ROS scavenging. We hypothesized that a ZIF-8 exoskeleton would protect SOD and CAT (SOD@CAT@ZIF-8), improve their stability, and overcome the current barriers to alleviate ROS-mediated renal damage.

**SCHEME 1:**
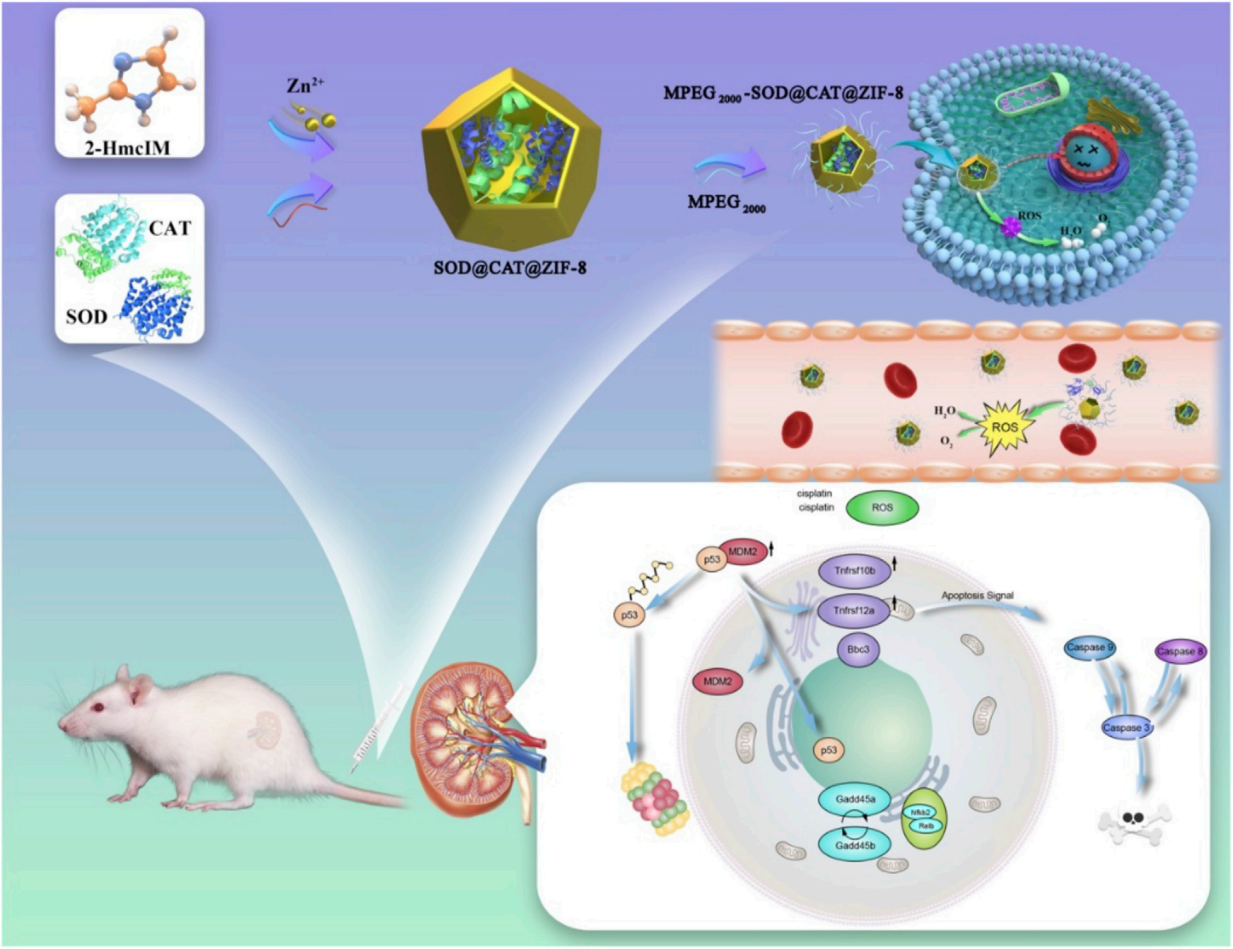
Schematic overview of PSCZ and their ROS scavenging application through a combination of SOD and CAT.

In the present study, we aim to develop a MOF encapsulating key antioxidant enzymes using a nanoscale ZIF-8 exoskeleton scaffold containing SOD and CAT (SOD@CAT@ZIF-8). This platform was further functionalized by anchoring MPEG_2000_-COOH to the surface of the MOF complex, yielding more stable and biocompatible composites (Scheme 1). Co-delivery of SOD and CAT enzymes can also be achieved using technology by linking the enzymes into enzyme complexes, followed by *in situ* free-radical polymerization. Different from these previous arts, this work uses a zeolitic imidazolate framework-8 to form enzyme–polymer nano complexes followed by an MPEG to encapsulate the nano complexes under mild conditions, resulting in the formation of multiple-enzyme nanoparticles. Relative to free CAT and SOD, this integrated MPEG_2000_-SOD@CAT@ZIF-8 (PSCZ) platform exhibited, more significant SOD and CAT enzymatic efficiency *in vitro* and was found to enhance ROS scavenging *in vivo,* protecting mice against AKI-associated oxidative renal tissue damage. Together, our data show MOF platforms incorporating integrated enzyme cascades and offer great promise for the *in vivo* biomedical treatment of AKI.

## 2 Results and Discussion

### 2.1 Synthesis and Characterization of PSCZ

**FIGURE 1:**
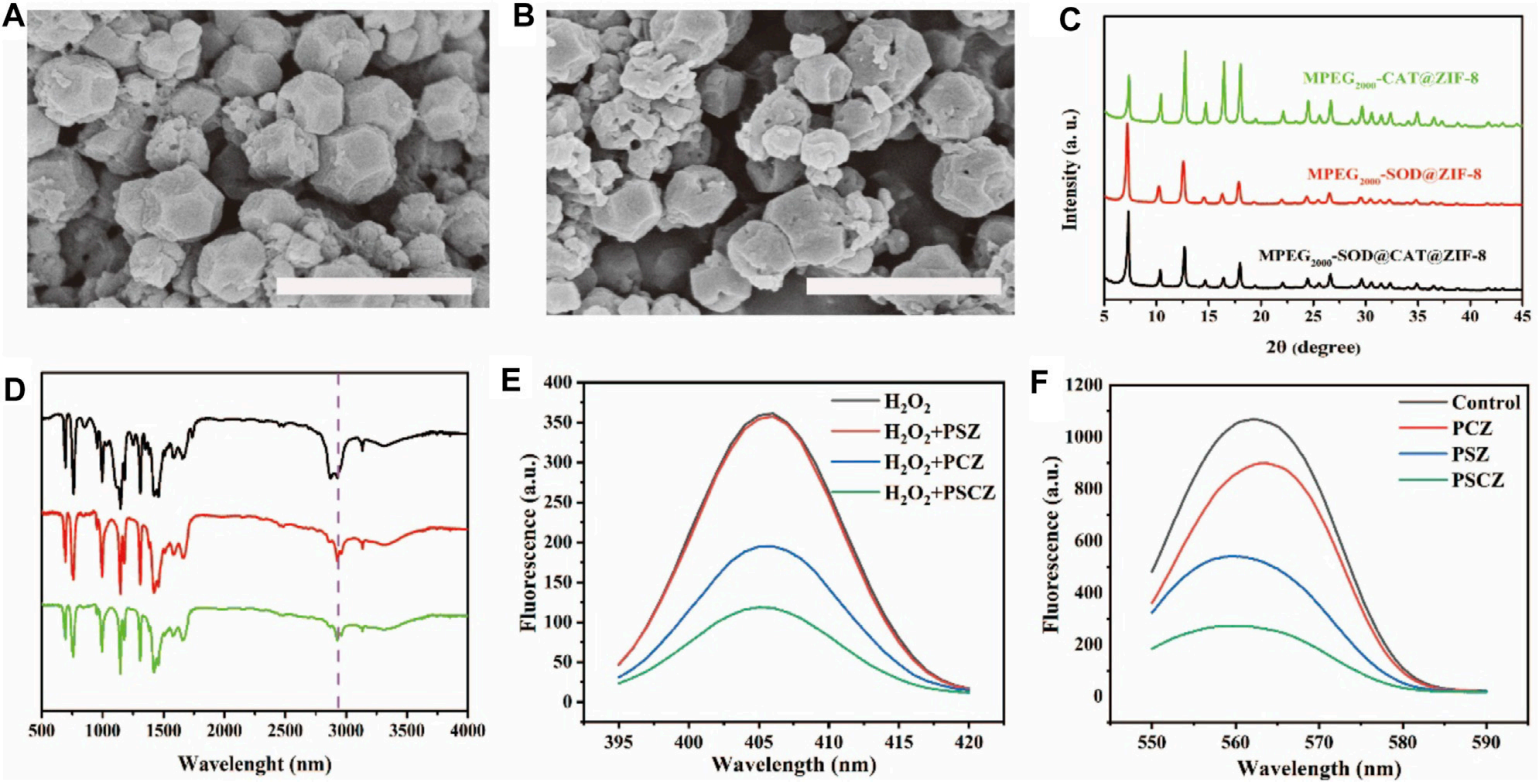
**(A,B)** SEM images of SOD@CAT@ZIF-8 and PSCZ following rinsing using dH_2_O; scale bar = 1 μm; **(C)** Powder X-ray diffraction (PXRD) patterns of PSZ, PCZ, and PSCZ; **(D)** Fourier transform infrared spectra (FT-IR) of PSZ (green), PCZ (red), and PSCZ (black); **(E)** CAT-like activity for PSZ, PCZ, and PSCZ; **(F)** Fluorescence spectra for SOD-like activity of PSZ, PCZ, and PSCZ with concentration (100 μg/ml).

As shown in Scheme 1, the native enzyme could be embedded in a porous nanomaterial ZIF-8, in which Zn (OAc)_2_, 2-HMeIM, SOD, and CAT were incubated for 5 min at room temperature. The morphology of composite SOD@CAT@ZIF-8 and PSCZ was characterized by SEM (Figure 1A). The SEM image showed that SOD@CAT@ZIF-8 and PSCZ both possessed uniform rhombic dodecahedral morphology similar to blank ZIF-8. As shown in Figure 1B, the PXRD pattern of MPEG_2000_-SOD@ZIF-8 (PSZ), MPEG_2000_-CAT@ZIF-8 (PCZ), and PSCZ both exhibited peaks matching well with the simulated ZIF-8 suggesting that ZIF-8 frameworks were formed in the biomimetic mineralization, and the structural integrity of ZIF-8 could be kept in the presence of SOD and CAT. FT-IR spectra of PSZ, PCZ, and PSCZ characteristic with MPEG_2000_-COOH peaks at 2,865–2,875 cm^−1^ that were respectively attributable to -COOH and -OCH_3_, indicating the successful conjunction of MPEG_2000_.

In order to prove that the SOD enzyme and CAT enzyme are encapsulated in the same crystal with ZIF-8, we used fluorescein isothiocyanate (FITC) to label SOD and Rhodamine 6G to label CAT. Detect the fluorescence spectra of the mixture (denoted as SOD+CAT) and PSCZ separately. As shown in Supplementary Figure S1, under an excitation wavelength of 450 nm, both the samples show emission at 520 nm attributed to FITC, whereas PSCZ exhibits an extra emission at 580 nm attributed to Rhodamine 6G, showing that the SOD enzyme and CAT enzyme are encapsulated in the same crystal of ZIF-8.

We further investigated the capacity to scavenge various free radicals of PSCZ, PSZ, and PCZ. Among biologically relevant ROS, H_2_O_2_ is of greatest importance because of its membrane permeability, longer half-life than O^2−^ and ^•^OH, and consequently highest intracellular concentration. CAT can catalyze the decomposition of H_2_O_2_ into H_2_O and O_2_, protecting cells and tissues from oxidative damage. The CAT-like activity of PCZ, PSZ free enzyme, and PSC cascade enzyme was investigated by recording the changes in absorbance of H_2_O_2_. We add xanthine oxidase, horseradish peroxidase, water-soluble tetrazolium salt (WST-1), and amplex red are added to enzyme solutions containing PSZ, PCZ, or PSCZ. Xanthine oxidase catalyzes the oxidation of WST-1, generating superoxide that is converted to H_2_O_2_ through the mediation of SOD. Meanwhile, mediated by horseradish peroxidase and H_2_O_2_, amplex red is converted to resorufin, a fluorescence probe for the H_2_O_2_ concentration of the solution. As shown in Figure 1E, the lower the absorbance, the stronger the scavenging ability of H_2_O_2_. The CAT-like activity of the PSCZ showed the best results within absorbance at 405 nm. SOD-like enzymes can treat a variety of oxidative stress-related diseases. The SOD-like ability of the PSCZ to eliminate O_2_^•−^ was measured using formazan, produced by the reaction of O_2_^•−^ and nitrogen blue tetrazole. The specific absorption of formazan was measured at 560 nm. The absorbance of formazan by PCZ, PSZ and PSCZ was detected at the same concentration, and the absorbance of PSCZ was far lower than PCZ and PSZ. The results showed that PSCZ had superior SOD-like enzyme activity.

Natural enzymes often exhibit intrinsic shortcomings, such as low operational stability, temperature, and pH sensitivity. Hence, the thermal and pH stabilities of PSCZ were investigated and compared with those of the natural enzyme SOD and CAT. The results (Supplementary Figures S2, S3) suggested that the stabilities of PSCZ when exposed to pH and temperature variations were significantly greater than those of natural SOD and CAT. These results show the increase in enzyme activity after biomimetic mineralization.

### 2.2 Assessment of the Ability of PSCZ to Scavenge Reactive Oxygen Species *In Vitro*

**FIGURE 2:**
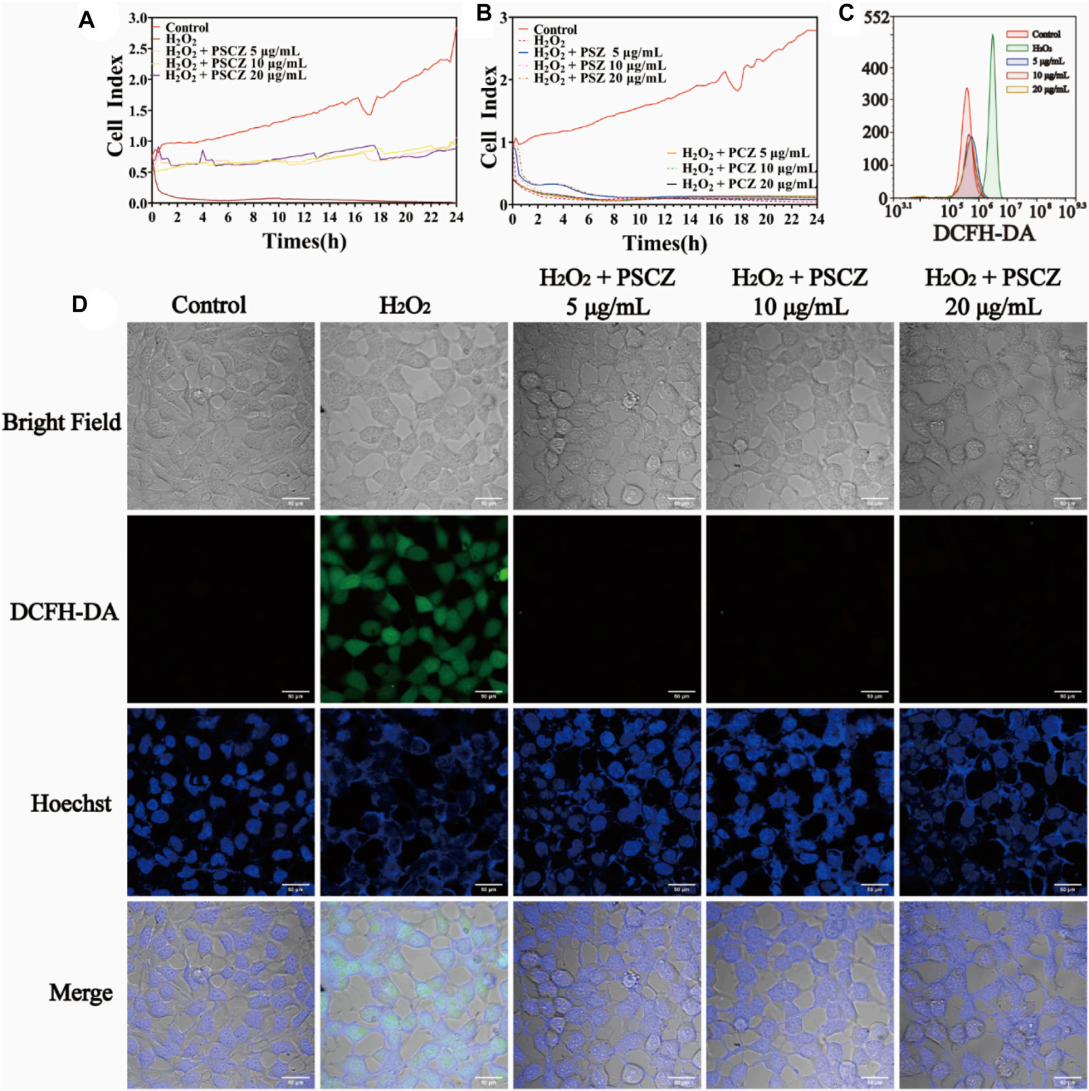
Assessment of the *in vitro* ROS scavenging activity of PSCZ. **(A,B)** An xCELLigence (RTCA) platform was used to assess HEK293 cell treatment following injured with H_2_O_2_ (200 μm) and select free enzymes **(A)** or PSCZ **(B)**. **(C)** ROS levels were assessed in HEK293 cells treated with select concentrations of PSCZ and H_2_O_2_ (200 μm). **(D)** Representative images of HEK293 cell ROS staining (green) in select treatment groups, with nuclei being stained using DAPI (blue).

The kidney is vulnerable to damage from ROS because it receives approximately 25% of the blood supply. The imbalance of ROS, referred to as oxidative stress, is detrimental to renal tubules and ultimately leads to AKI. Given the promising properties of PSCZ particles as stable, biocompatible antioxidant agents, we next assessed their ROS scavenging activity *in vitro* relative to that of free CAT and SOD. Given that AKI pathogenesis is associated with oxidative damage to the renal tubules, we used the HEK293 cell line model to assess the ability of PSCZ to protect against ROS-mediated injury (Pavlakou et al., 2017). H_2_O_2_ reacts to generate free radicals responsible for the cause of cell damage. Therefore, the ability of PSZ, PCZ, free SOD, free CAT, ZIF-8 and PSCZ to reduce the levels of H_2_O_2_ and ROS production was investigated in HEK293 cells. Cell viability test reveals PSC, PCZ, free SOD, free CAT, and ZIF-8 had no protective effect on cells under the stimulation of H_2_O_2_ (200 μm) injury, while PSCZ significantly improves cell viability (Supplementary Figure S4; Supporting Information). In order to further compare the cell protective effect of PSCZ, we additionally used an xCELLigence (RTCA) instrument to monitor the survival and growth of these cells in a quantitative, real-time, dynamic manner. This assay approach is superior to traditional endpoint-based viability assays that assess membrane permeability (Yah and Simate, 2020), enabling the assessment of cell survival in a label-free and automated manner throughout a given experimental period (Ceti̇n and Topcul, 2019). RTCA approach was used to measure the survival and growth of cells over a 24 h period following injury with H_2_O_2_ and these different nanoenzymes preparations. PSCZ showed superior antioxidant capacity at 5 μg/ml than PSZ and PCZ even at a higher (20 μg/ml) concentration (Figure 2A). We then injured HEK293 cells with H_2_O_2_ to induce oxidative stress to evaluate the ability of PSCZ to protect against oxidative damage (Figure 2B). While the viability of H_2_O_2_-treated cells was ∼50%, PSCZ treatment was associated with a significant increase in these cells’ survival because of the observed reductions in intracellular ROS production and the consequent preservation of mitochondrial functionality (Figure 2B).

We also used the DCFH-DA fluorescent probe to assess intracellular ROS levels to further confirm whether PSCZ could suppress H_2_O_2_-induced ROS generation in HEK293 cells. As shown in Figure 2D, it shows that H_2_O_2_ injury of HEK293 cells stimulates ROS production, while in PSCZ/H_2_O_2_-injured cells reduced ROS production was observed. These results show that, compared with other groups, PSCZ can effectively reduce the generation of free radicals mediated by H_2_O_2_. PSCZ is safe for *in vitro* and *in vivo* applications. This finding was further confirmed via flow cytometry (Figure 2C), revealing that PSCZ could reliably suppress H_2_O_2_-mediated free radical generation.

Mitochondrial oxidative phosphorylation is the primary mechanism whereby most cells produce ATP (Xu et al., 2019). Given that renal PTECs lack glycolytic activity and are under high metabolic energy demands, these cells exist under conditions of ischemia and hypoxia (Yu et al., 2018). This increases the susceptibility of these renal tubular epithelial cells to acute renal injury. Mitochondria are also central regulators of intracellular apoptotic signaling, with the loss of mitochondrial membrane potential (Δψm) being an early apoptotic indicator (Sastre et al., 2000). Therefore, we assessed the impact of PSCZ on HEK293 cell Δψm using a JC-1 staining approach, with a higher red/green JC-1 ratio indicative of lower levels of mitochondrial dysfunction. We observed a significant decrease in the Δψm of these cells following H_2_O_2_ injury (Supplementary Figure S5), whereas PSCZ treatment yielded a JC-1 fluorescence ratio comparable to that in control cells, suggesting that PSCZ can preserve mitochondrial membrane integrity.

**FIGURE 3:**
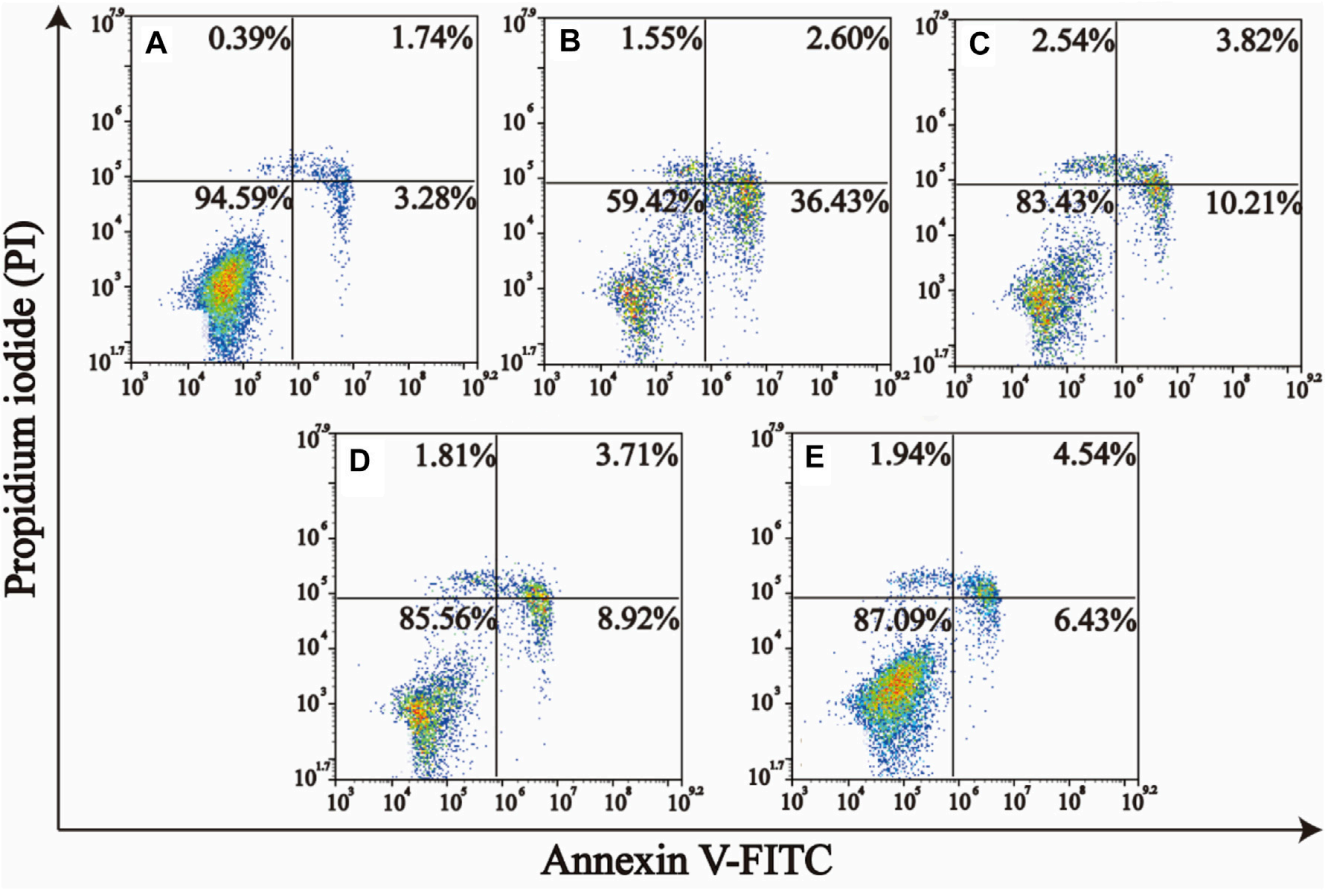
PSCZ protects HEK293 cells against oxidative damage induced by H_2_O_2_. AnnexinV–fluorescein isothiocyanate (FITC)/PI dual-staining was used to assess the impact of various PSCZ concentrations on apoptotic cell death in HEK293 cells that had been treated for 24 h with H_2_O_2_ (200 μm). **(A)** Control group; **(B)** H_2_O_2_ injury (200 μm); **(C)** H_2_O_2_ (200 μm)+PSCZ 5 μg/ml; **(D)** H_2_O_2_ (200 μm)+PSCZ 10 μg/ml; **(E)** H_2_O_2_ (200 μm)+PSCZ 20 μg/ml.

Excess ROS generation can initiate apoptotic signaling cascades within cells (Ratliff et al., 2016; Meng et al., 2018). Given the acute sensitivity of renal tubule cells to oxidative damage, we next assessed the ability of PSCZ to facilitate *in vitro* ROS scavenging activity in HEK293 cell model. When the death of these cells was assessed *via* flow cytometry following Annexin V/PI staining, we found that H_2_O_2_ injured (200 μm) increased the frequency of apoptotic HEK293 cells to 36.43%, relative to control cells (3.28%) (Figure 3). In contrast, cells treated with PSCZ exhibited significantly lower rates of apoptotic death (6.43%). These findings thus confirmed that PSCZ could protect against oxidative damage in HEK293 cells *in vitro.*

### 2.3 PSCZ Effectively Facilitates *In Vivo* ROS Scavenging

Given the promising *in vitro* results detailed above, we next explored the *in vivo* utility of our MOF antioxidant platform using a murine cisplatin-induced AKI (CP-AKI) model system (Rewa and Bagshaw, 2014). Cisplatin (CDDP) is a widely used chemotherapeutic drug that accumulates within renal proximal tubule epithelial cells (PTECs), resulting in localized renal inflammation, damage, apoptotic death, and AKI that can manifest as CDDP-associated nephrotoxicity (Manohar and Leung, 2018). The pathogenesis of CP-AKI has been linked to ROS production and consequent acute tubular apoptosis (Ozkok and Edelstein, 2014). Thus, this CP-AKI model system was selected for further analyses of the relative safety and *in vivo* antioxidant activity of PSCZ.

**FIGURE 4:**
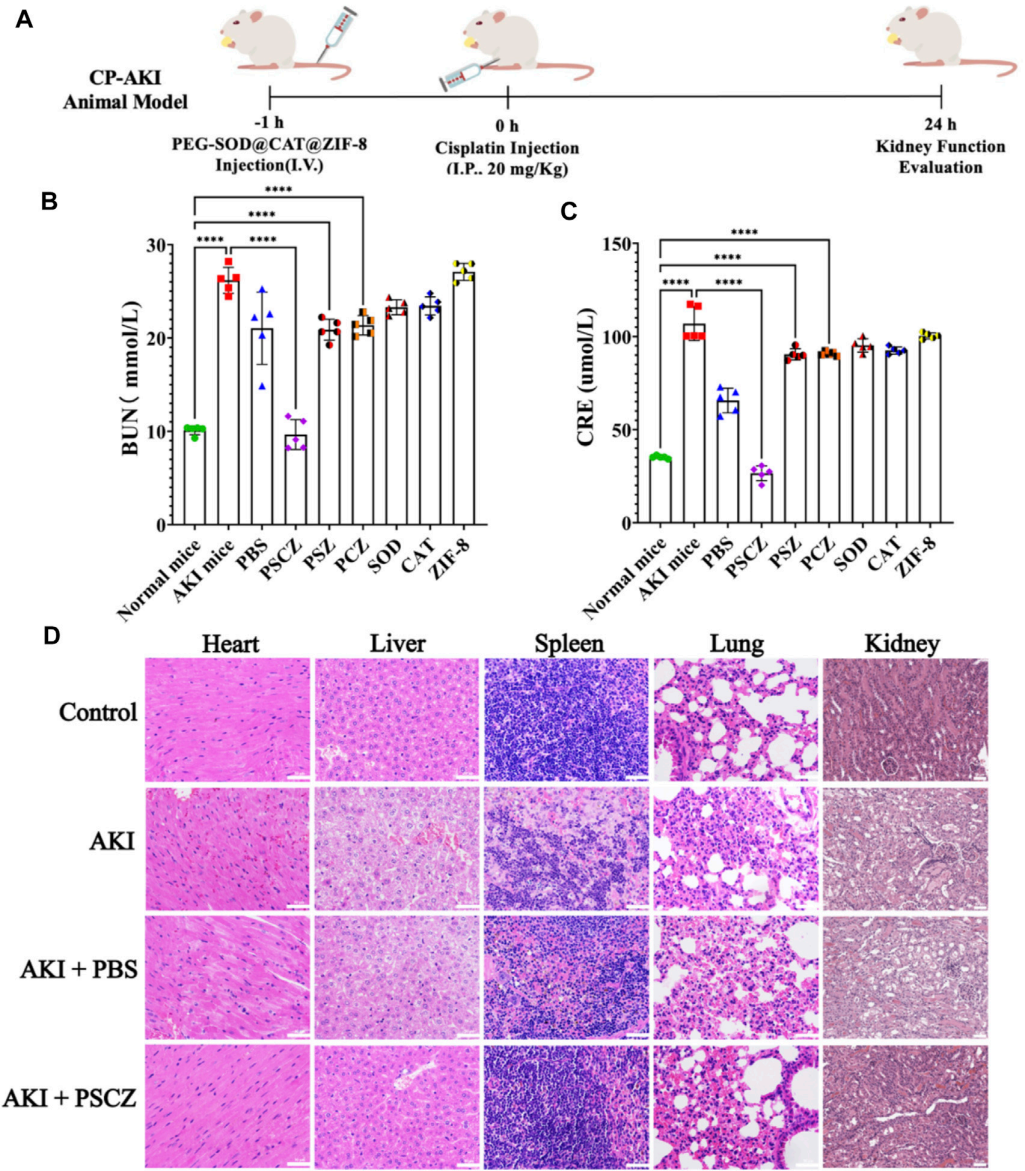
Assessment of the biocompatibility of PSCZ *in vivo*. **(A)** CP-AKI modeling approach. **(B)** CRE levels in CP-AKI mice were measured at 24 h following select treatments. **(C)** BUN levels in CP-AKI mice were measured at 24 h following select treatments. **(D)** Tissues from select treatment groups were assessed following H&E staining.

The construction of the CP-AKI animal model is consistent with previous published articles (Ni et al., 2018; Sun et al., 2018; Rosenkrans et al., 2020; Yu et al., 2020). Briefly, CP-AKI mice were established as in prior reports by intraperitoneally injecting these animals with CDDP (20 mg/kg) and then measuring serum BUN and CRE levels after 24 h to gauge renal function (Bolisetty et al., 2016), given that these compounds accumulate in the blood in renal damage. To evaluate the effect of treatment *in vivo*, mice in corresponding treatment groups were intravenously treated with PSZ, PCZ, free SOD, free CAT, ZIF-8, and PSCZ 1 h before CDDP administration (Figure 4A), as *Liu*’s research (Liu et al., 2020). Blood serum samples in each group were analyzed for CRE and BUN concentrations. CRE and BUN are nitrogenous waste products and accumulate in the blood when kidney function has been impaired. As a result, CRE and BUN are widely used in the clinic to assess kidney function. In CP-AKI mice, CRE and BUN levels were significantly higher in CP-AKI mice than in healthy controls (Figures 4B,C), consistent with the successful induction of CDDP-mediated kidney damage in these animals. Saline infusions are commonly administered to patients before and during CDDP treatment (Bolisetty et al., 2016) infusions exhibit poorly understood renoprotective activity against CDDP-induced nephrotoxicity. However, CP-AKI will manifest in roughly 30% of treated patients (Sharp et al., 2016). Our mouse model system observed comparable findings, as only slight reductions in CRE and BUN levels were observed in CP-AKI mice treated with PBS, with these levels being significantly higher than those in control animals. In contrast, the treatment of CP-AKI mice with PSCZ resulted in significant reductions in CRE and BUN levels to concentrations comparable to those in healthy control mice, however, free SOD, free CAT, PSZ, and PCZ mice in other groups had no significant therapeutic effect. These data underscored the therapeutic utility of PSCZ as a safe and effective tool for treating CP-AKI in mice.

We additionally isolated renal tissue sections from these treated mice and stained them with H&E stain to assess treatment-related changes in tissue pathology. Renal casts can arise due to denatured protein precipitation in kidney tubules and are often considered being an indicator of kidney disease. Similarly, inflammatory cell infiltration and the formation of vacuoles within renal tubules can be visualized to detect and gauge the severity of the renal inflammatory injury. While many casts were clear in the renal tissue samples from CP-AKI model mice (Figure 4D; Supplementary Figure S8), relatively few were detectable in AKI model animals that had been administered PSCZ, consistent with the ability of these nanoparticles to preserve the integrity of renal tissues better. The H&E staining of other tissues shows that PSCZ may have a therapeutic effect on inflammation of these tissues caused by cisplatin, but it is not within the scope of this study (Supplementary Figure S8).

To further assess the ability of PSCZ to suppress renal ROS generation, we next used the fluorescent ROS probe DCFH-DA to stain kidney tissue sections from CP-AKI mice in our different treatment groups and then imaged these tissues via laser scanning confocal microscopy. This approach revealed that PSCZ administration significantly inhibited renal ROS accumulation in our AKI model mice (Supplementary Figures S6, S7).

**FIGURE 5:**
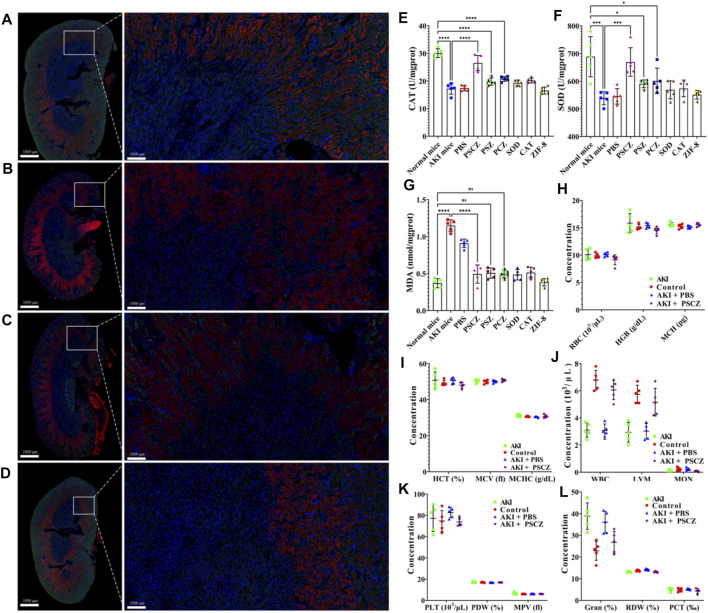
Analysis of renal biomarker expression and ROS accumulation. **(A–D)** TNF-α and IL-1β levels in mice from the treatment groups were detected via immunofluorescent staining. **(A)** Control; **(B)** CP-AKI; **(C)** CP-AKI+PBS; **(D)** CP-AKI+PSCZ; **(E)** CAT levels in renal homogenates; **(F)** SOD levels in kidney homogenates; **(G)** MDA levels for mice in the groups; **(H–L)** The hematological parameters were assessed in murine treatment groups at 24 h post-PSCZ injection.

Tumor necrosis factor-alpha (TNF-α) and interleukin-1β (IL-1β) are key inflammatory cytokines and drivers of apoptotic cell death in cisplatin-induced renal injury (Martindale and Holbrook, 2002; Schieber and Chandel, 2014). Excess ROS production by cells under inflammatory conditions can induce additional proinflammatory cytokine production and promote further immune cell infiltration and renal damage. IL-1β can further amplify these inflammatory processes through feedback mechanisms (Ozkok and Edelstein, 2014). Renal tissue samples from treated mice were collected and analyzed to reveal the underlying mechanism of PSCZ. Immunofluorescence results showed that IL-1β and TNF-α levels were comparable in the PSCZ-treated and control groups, suggesting these nanoparticles did not induce direct inflammation in mice at the used concentrations and can reduce inflammation-induced kidney damage (Figures 5A–D). Notably, in AKI model mice, PSCZ administration suppressed TNF-α and IL-1β expression relative to untreated model controls. We additionally assessed SOD and CAT levels in renal homogenates from these mice (Figures 5E,F), to confirm that these antioxidant enzymes were successfully delivered to damaged kidney tissue (Hao et al., 2019). Excessive ROS levels can cause SOD and CAT depletion, as was clear in CP-AKI and PBS-treated mice. In contrast, PSCZ resulted in renal SOD and CAT levels similar to those in healthy control animals. It was confirmed that PSCZ was successfully delivered to the injured renal tissue and alleviated renal inflammatory injury.

To further test the therapeutic utility of PSCZ, we next measured malondialdehyde (MDA) levels and blood biochemical parameters to evaluate renal excretory function. MDA analysis results revealed that both CP-AKI mice and PBS-treated AKI mice exhibited elevated MDA levels consistent with renal failure, while PSCZ administration lowered these levels consistent with the alleviation of AKI-related renal damage. Similar to other results, treated with PSZ, PCZ, free SOD, free CAT or ZIF-8 did not reduce MDA levels (Figure 5G).

Analyses of blood biochemical parameters from these mice showed that PSCZ treatment was not associated with obvious renal toxicity (Figures 5H–L), and hematological parameters in PSCZ-treated animals were comparable to those in healthy controls. These findings suggested that our PSCZ particles were highly biocompatible and useful as a tool for AKI treatment. For biocompatibility and biodistribution, we did not perform further repeated verification, because He et al. have conducted related research (Rosenkrans et al., 2020). However, it is essential to note that we only tested for acute toxicity associated with this therapeutic platform, and further research will be necessary to establish whether PSCZ is retained in the kidney beyond the 24 h time point, whether it is eliminated via renal metabolic processing, and whether it induces any long-term toxicity *in vivo.*

### 2.4 RNAs-seq Analysis Confirmed the Successful Construction of the Acute Kidney Injury Model and PSCZ Showed a Superior Protective Effect in the Acute Kidney Injury Mouse Model

In the current study, we set four different groups: control group with no treatment (brief as the control group), cisplatin-induced AKI group (brief as CP-AKI group), AKI group pre-treated with PSCZ (brief as the PSCZ+AKIgroup), and AKI group pre-treated with PBS (brief as the PBS+CP-AKI group). More detailed information is shown in the M&M section.

An average of 63.34 (range from 58.51 to 66.33) million raw reads were obtained for all samples. After removing low-quality reads, an average of 60.52 (range from 56.05 to 63.16) million clean reads were retained for further analysis. The raw data were uploaded to National Omics Data Encyclopedia (NODE) database (https://www.biosino.org/node) with the following accession number: OEP000285.

**FIGURE 6:**
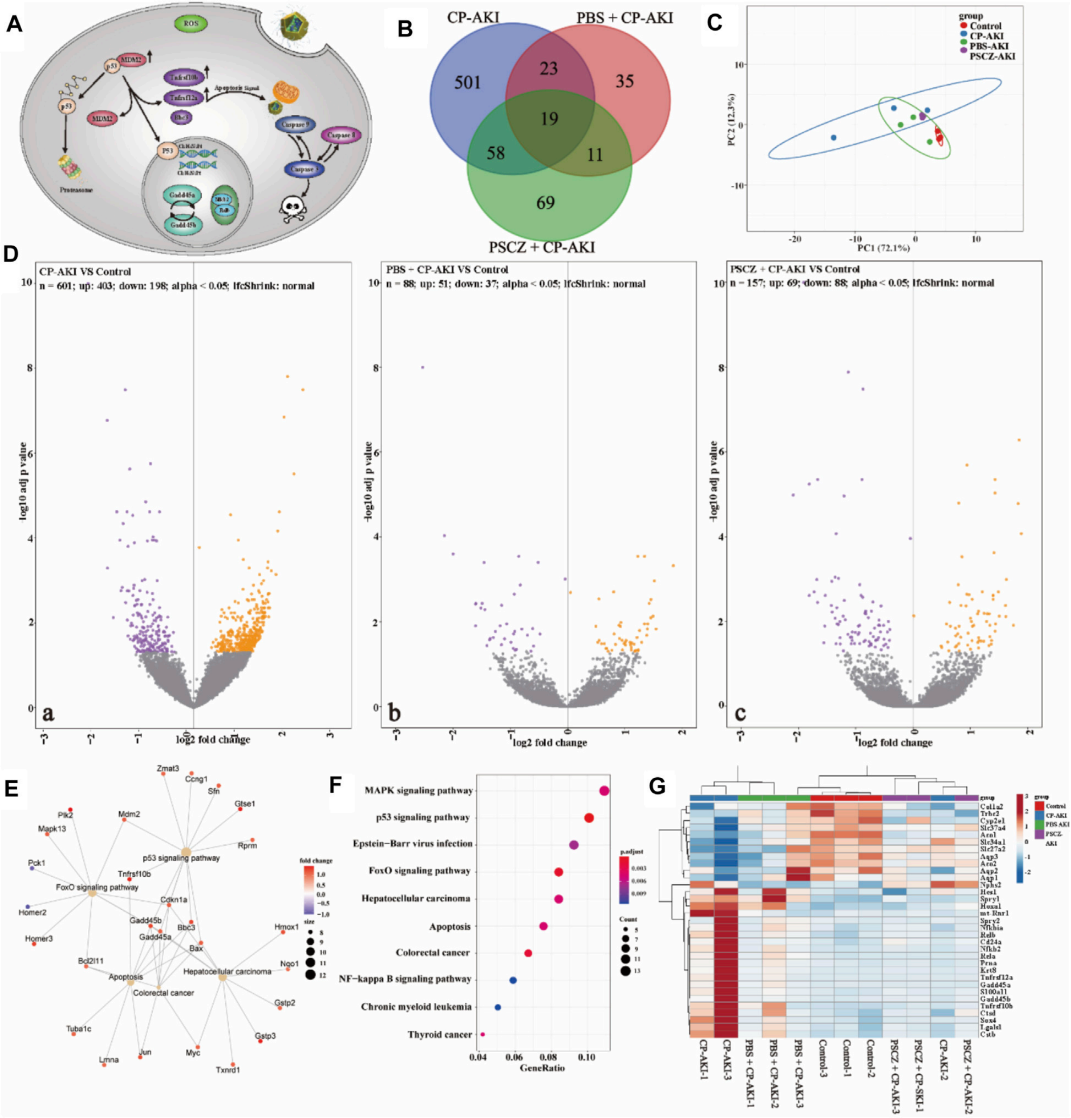
Results from transcriptome sequencing analyses. **(A)** Mechanism of the figure; **(B)** Venn diagram of the transcriptome profiles between CP-AKI, PBS +CP-AKI, PSCZ+CP-AKI groups; **(C)** Principal component analysis (PCA) was performed based on differentially expressed genes from the kidneys of two groups. Each data point corresponds to the PCA analysis of each sample; **(D)** Volcano plots showing the identified upregulated and downregulated genes in each group; **(E)** Gene mapping of differentially expressed genes involved in oxidative stress; **(F)** KEGG pathway enrichment analysis of the identified differentially expressed genes; **(G)** Up-regulated and down-regulated genes involved in the oxidative stress after PSCZ treatment.

To further elucidate the underlining therapeutic mechanisms, CP-AKI was chosen as the representative disease model for further transcriptome analysis. DESeq2 identified 601 DEGs, 157 DEGs, and 88 DEGs for the CP-AKI group, CP-AKI+PSCZ group, and CP-AKI+PBS group compared with the control group, respectively (Figure 6D). An unguided principal component analysis (PCA) of the data revealed different transcriptome profiles between PSCZ and PBS-treated AKI mice kidneys (Figure 6C). The Venn diagram in Figure 6B showed many differentially expressed genes were produced in the CP-AKI group compared with the control group, and the differentially expressed genes were decreased in the PSCZ treatment group.

We also investigated the impact of PSCZ on gene expression related to growth arrest and DNA damage response, apoptosis, oxidation-reduction, etc. (Figure 6G). In brief, the PSCZ treatment could reverse the impact of cisplatin-induced AKI in the mouse model.

DEGs in the CP-AKI group were enriched in the p53 signaling pathway, FoxO signaling pathway, apoptosis-related pathways, etc (Figures 6E,F; Supplementary Figure S9). This is consistent with previous research. Cisplatin can interfere with DNA replication and DNA repair mechanisms, cause DNA damage, and induce apoptosis in cells (Dasari and Bernard Tchounwou, 2014). Cisplatin can also increase cellular ROS and induce endoplasmic reticulum (ER) stress, contributing to cisplatin toxicity. Cell death and ER stress are characteristic of cisplatin-induced AKI (Sharp et al., 2016; Sharp and Siskind, 2017). P53 signaling is an early stage response to cisplatin toxicity in renal cells. Cisplatin caused renal cells to initiate the p53 dependent DNA repair pathway. If ROS continues to be overproduced in renal cells, the intrinsic apoptotic pathway will be activated. In the early stage, when cisplatin interferes with DNA replication and DNA repair mechanism, nano inhibits the excessive production of ROS caused by cisplatin, promotes the initiation of DNA repair pathway, and alleviates renal cell damage (Figure 6A; Supplementary Figure S10).

In the current study, the kidney proximal nephron tubule segment injury marker *Havcr1* (also known as *Kim1*) slightly changed (log_2_FC = 0.42, *adj. P* = 0.437) CP-AKI group compared with the control group. This is probably due to the kidneys were harvested in the very early stage of AKI. This is consistent with a previous study that cisplatin-induced AKI mainly caused proximal nephron tubule segment injury in the very early stage (Bolisetty et al., 2016). *Bax*, *Bak1* are important signals in the apoptosis cascade and normally act on the mitochondrial membrane to promote permeabilization and release of cytochrome C and ROS. In CP-AKI group, *Bax* and *Bak1* significantly increased (log_2_FC = 1.16, adj. *P* = 0.024; log_2_FC = 0.99, adj. *P* = 0.048, respectively). This means that ROS level increased by cisplatin. *Aco1* and *Cyp2e1*, which are important oxidation-reduction enzymes, significantly decreased (log_2_FC = −0.74, adj. *P* = 0.007; log_2_FC = −1.03, adj. *P* = 0.026, respectively). This indicated that the normal function of oxidation-reduction was compromised. *Gadd45a* and *Gadd45b*, which are growth arrest and DNA damage response genes, also increased (log_2_FC = 1.13, adj. *P* = 0.045; log_2_FC = 1.29, adj. *P* = 0.024, respectively). This indicated that kidney cells responded to cisplatin-induced DNA damage by cell cycle arrest and initiated the DNA repair system, in which case, the p53 signaling pathway. *Tnfrsf12a* and *Tnfrsf10b* (also known as *Dr5*), which belong to tumor necrosis factor receptor superfamily and transduces apoptosis signals, significantly increased in CP-AKI group (log_2_FC = 1.50, adj. *P* =0.003; log_2_FC = 1.45, adj. *P* =0.006, respectively). *Caspase8* also significantly increased in CP-AKI group (log_2_FC = 0.74, adj. *P* =0.044). This indicated that cisplatin-induced intrinsic apoptosis through *Caspase8* by upregulating *Tnfrsf10b* in the CP-AKI group. The results demonstrated that the apoptosis process was initiated in the CP-AKI group after 24 h of being treated with cisplatin. *Bcl10* contains a caspase recruitment domain (*CARD*) and has been shown to induce apoptosis and activate NF-κB. *Bcl10* slightly increased in CP-AKI group (log_2_FC = 0.70, adj. *P* =0.159).

However, this process is reversible if cell oxidative stress can be alleviated. In PBS and PSCZ pre-treated group, *Bax* and *Bak1* showed the tendency to return to normal level (*Bax*: log_2_FC = 0.89, adj. *P* = 0.260; log_2_FC = 0.52, adj. *P* = 0.774, respectively; *Bak1*: log_2_FC = 0.39, adj. *P* = 0.836; log_2_FC = 0.11, adj. *P* = 1.000, respectively), *Tnfrsf12a* and *Tnfrsf10b* also showed the tendency to return to normal level (*Tnfrsf12a:* log_2_FC = 0.54, adj. *P* = 0.500; log_2_FC = 0.13, adj. *P* = 1.000, respectively; *Tnfrsf10b:* log_2_FC = 0.99, adj. *P* = 0.177; log_2_FC = 0.42, adj. *P* = 0.860, respectively) compared with CP-AKI group. This indicated that renal cell oxidative stress was significantly ameliorated, and cell apoptosis was repressed. *Gadd45a* and *Gadd45b* also showed the tendency to return to normal level in PBS and PSCZ pre-treated AKI mouse models (*Gadd45a*: log_2_FC = 0.35, adj. *P* = 0.897; log_2_FC = −0.04, adj. *P* = 1.000, respectively; *Gadd45b*: log_2_FC = 0.38, adj. *P* = 0.862; log_2_FC = 0.33, adj. *P* = 0.957, respectively). This indicated cell cycle arrest was also alleviated in both groups. Tadagavadi et al. also reported that PBS had protective effects in AKI models (Tadagavadi and Reeves, 2010). This is consistent with the findings in current study. In the current study, PSCZ showed superior protective effect than PBS.

## 3 Materials and Methods

### 3.1 Materials

Zn(NO_3_)_2_•6(H_2_O), 2-methyl imidazole (2-HmeIM), polyvinylpyrrolidone (PVP), SOD, CAT, and Fluorescein isothiocyanate (FITC) were purchased from Sigma-Aldrich and used as received. Methoxy-PEG-acid (MPEG_5000_-COOH) and NH_2_-PEG_2000_-COOH were purchased from Ponsure Biological. Phosphate buffer solution (PBS) was purchased from Thermo-Fisher (Waltham, MA, United States). LysoTracker Green and Reactive Oxygen Species Assay Kit were purchased from Beyotime (China). All the aqueous solutions were prepared using purified deionized (DI) water purified with a purification system (Direct-Q3, Millipore, United States). The other solvents used in this work were purchased from Sinopharm Chemical Reagent (China) and Aladdin-Reagent (China).

### 3.2 Construction of SOD@CAT@ZIF-8 Through Biomimetic Mineralization

Typically, 4 ml aqueous solution containing HMeIM (990 mg, 12 mM) was mixed with 4 ml of Zn(NO_3_)_2_•6(H_2_O) solution (52 mg, 0.175 mM), 400 µl of SOD (10 mg) and CAT (10 mg) solution. The mixture was incubated at room temperature for 5 min, and then the centrifuged pelletes were collected and washed with deionized water. The white solids were subsequently dried at room temperature to obtain the composite SOD@CAT@ZIF-8. The SOD and CAT loading in ZIF-8 were calculated using the following formula:

Protein loading (%) = [(A-B)/A] × 100%

A and B represented the amount of protein in the initial solution and washing solution, measured based on the BCA assay.

### 3.3 Synthesis of PSCZ

MPEG_2000_-COOH was connected to the surface of SOD@CAT@ZIF-8 through a coordination bond between -COOH group and the Zn ion to prepare PMs. Briefly, pre-formed CAT@SOD@ZIF-8 was dispersed in an MPEG_2000_-COOH solution of 10 mg/ml using deionized water as the solvent. The mixture was sonicated for 10 min and stirred at room temperature for 48 h to form a PSCZ suspension. The white product was collected by centrifugation, followed by washing three times with deionized water and drying at 35°C under vacuum.

### 3.4 Superoxide Anion Scavenging With PSCZ

The superoxide anion scavenging activity was assessed with a SOD assay kit (Solarbio, China). The MPEG_2000_-SOD@CAT@ZIF-8 (PSCZ), MEPG_2000_-SOD@ZIF-8 (PSZ), MPEG_2000_-CAT@ZIF-8 (PCZ) concentrations (100 μg/ml) test was performed according to the instructions provided with the kit. The absorbance at 560 nm was measured using a quartz colorimetric dish reader after incubating at 37°C for 30 min.

### 3.5 PSCZ Scavenger Hydrogen Peroxide

The superoxide anion scavenging activity was assessed with a CAT assay kit (Solarbio, China). The PSCZ, PSZ, PCZ concentrations (100 μg/ml) test was performed according to the instructions provided with the kit. The absorbance at 405 nm was measured using a quartz colorimetric dish reader after incubating at room temperature for 10 min.

### 3.6 Viability of Cells Protected by PSCZ From Oxidative Stress

Human embryonic kidney 293 (HEK293) was cultured in Dulbecco’s Modified Eagle Medium (DMEM) (1% penicillin/streptomycin and 10% fetal bovine serum) at 37°C under 5% CO_2_. Using an RTCA E-Plate, HEK293 cells were seeded at a concentration of 1 × 10^4^ cells per well and incubated for 24 h. Next, PSCZ, PSZ, and PCZ dispersed in the cell culture medium at various concentrations (5, 10, 20 μg/ml) were added to the wells and incubated for 1 h H_2_O_2_ was then added to the cells to a final concentration of 200 μm and incubated for 24 h. Cells with no treatment and treated with only H_2_O_2_ were used as controls compared with only PSCZ, PSZ, and PCZ. The standard RTCA analysis is used to measure cell viability.

### 3.7 PSCZ Scavenging of Reactive Oxygen Species *in vitro*

2′,7′-Dichlorofluorescin diacetate (DCFH-DA, D6883, Sigma-Aldrich, United States), an oxidation-sensitive fluorescent dye, was used to detect the intracellular ROS level, according to the literature. Briefly, DCFH-DA is a non-fluorescent chemical compound that could freely diffuse through the cell membrane and be hydrolyzed by intracellular esterase to DCFH. The intracellular ROS could oxidize the non-fluorescent DCFH to fluorescent DCF. Therefore, the quantity of intracellular ROS is correlated with the fluorescent intensity of DCF. After incubation with H_2_O_2_ for 24 h, cells were gently rinsed thrice with a serum-free medium to remove the free PSCZ. Then, a final concentration of 10 μM of DCFH-DA in serum-free medium was added to the cells and incubated in the dark at 37°C for 30 min. Afterward, the cells were washed with serum-free medium thrice to remove the unloaded DCFH-DA probe, then were scanned using a laser confocal microscope (Zeiss LSM780, Germany). Flow cytometry analysis was used to quantify the intracellular ROS levels.

Next, cells seeded in six-well plates were stained with Annexin V-FITC apoptosis detection kit (A211-01, Vazyme, China) to detect the ratio of apoptotic and necrotic cells. Briefly, HEK293 cells in a well were collected, washed with cold PBS, and re-suspended in 195 μl binding buffer after incubation with H_2_O_2_ for 24 h. Then, 5 μl Annexin V-FITC and 10 μl PI were sequentially added to the cell suspension and incubated at room temperature in the dark for 15 min. After that, cells were analyzed by the Flow cytometer (NovoCyte, Agilent, United States). At least 50,000 cells were analyzed in each sample.

Finally, we used the mitochondrial membrane potential detection kit JC-1 (C2006, Beyotime, China) probe method to detect mitochondrial membrane potential changes. HEK293 cells were seeded separately into confocal laser Petri dishes. Follow the instructions provided with the kit. After incubation with H_2_O_2_ for 24 h, cells were gently rinsed thrice with PBS to remove the free PSCZ. Then add 1 ml of JC-1 dye solution and mix well. Then the cells were incubated in an incubator at 37°C for 20 min. An appropriate amount of JC-1 staining buffer (1×) was prepared by adding 1 ml of JC-1 staining buffer (5×) to 4 ml of distilled water and placed in an ice bath during incubation. At the end of incubation at 37°C, the supernatant was aspirated, washed twice with JC-1 staining buffer (1×), and then 2 ml cell culture solution was added, which could contain serum and phenol red. Finally, using a laser confocal microscope (Zeiss LSM780, Germany).

### 3.8 PSCZ Scavenging of Reactive Oxygen Species *in vitro*

#### 3.8.1 Cisplatin-Induced Acute Kidney Injury Model

Female BALB/c mice aged 6–8 weeks were selected, all mice were received intraperitoneal injections of cisplatin (20 mg/kg). Twenty four hours after injections, the mice in each group were sacrificed to monitor the model development. The blood samples and renal tissues were obtained and analyzed. The mice were intravenously injected with PSCZ (15 mg/kg in 100 μl PBS) at 1 h after intraperitoneal injections of cisplatin for the treatment group.

#### 3.8.2 Treatment of Cisplatin-Induced-Acute Kidney Injury Mice

One hour after the AKI model induction, different treatments were performed on AKI model mice: group 1 was healthy mice (*n* = 5); group 2 was AKI mice (PBS, *n* = 5); group 3 was AKI mice treated with 1× PBS (*n* = 5); group 4 was AKI mice treated with PSCZ (15 mg/kg in 100 μl PBS, *n* = 5). After 24 h p.i, blood samples and tissues of major organs were collected, the treatment group was compared with healthy mice to evaluate kidney function.

#### 3.8.3 Kidney Function Test

After euthanizing the mice, blood samples were collected in lithium heparin tubes. The blood serum was collected after centrifuging the blood samples at 2,000 g for 15 min at 4°C. Finally, the samples were sent to the Laboratory Department of the First Affiliated Hospital of Zhengzhou University for analysis of blood urea nitrogen (BUN) levels and blood creatinine (CRE) levels.

#### 3.8.4 Hematoxylin and Eosin Staining of Kidney Sections

In the AKI model, mice were euthanized to harvest the kidneys at the time-point of interest. Next, the kidneys were fixed with paraformaldehyde (4% in PBS) and embedded in paraffin wax, sliced, and stained with H&E stain in the Laboratory Animal Center of Zhengzhou University.

#### 3.8.5 Analysis of the Therapeutic Effect in Renal Tissue

Kidneys from each group were snap-frozen and stored at −80°C until use. Kidney homogenates were prepared according to the protocols of different assays. SOD level and CAT level were assessed with a SOD assay kit and CAT assay kit (Sigma-Aldrich, United States). Kidney tissues of mice in each group were sliced using a frozen micro-slicer to evaluate ROS’s scavenging effect. Frozen kidney tissue slides (about 5 μm thickness) were washed with PBS. One of the slides was stained with 1 mM DCFH-DA for 30 min to detect superoxide formation, and another slide was stained with TNFα- and IL1β antibodies and DAPI to evaluate the development of inflammation in the tissues. Then a cover glass was applied to each slide using Vectashield mounting medium. Finally, a laser confocal microscope was used to observe the results.

#### 3.8.6 RNA Sequencing

Mice were sacrificed to harvest the kidneys. The kidneys were washed thrice with saline and snap-frozen in liquid nitrogen. Total RNA was extracted by using the TIANGEN Animal Tissue Total RNA Extraction Kit (DP419, TIANGEN, China). RNA integrity was verified by Agilent 4200 Bioanalyzer and quantified using ND-2000 (Nanodrop Technology). Cell rRNA was removed by using MGIEasy rRNA removal kit. Library construction was carried out using the MGIEasy RNA Library Prep Set (96 RXN) according to the manufacturer’s protocol. Libraries were visualized on the Agilent 4200 Bioanalyzer to check insert size and quantified by using the Qubit Fluorometers to determine the concentration. Libraries were pooled and loaded on the flow cell to run on MGISEQ-2000 sequencer as paired-end read for 150 cycles on each side.

### 3.9 Bioinformatics Analysis

#### 3.9.1 Transcriptome Sequencing and Mapping

Clean reads were mapped to Mus musculus reference genome (GRCm38) with STAR aligner (version 2.6.1d) (Dobin et al., 2013). Transcript-level gene expression was estimated by Salmon (version 0.14.1) (Patro et al., 2017). Transcript per million (TPM) values were calculated using tximport. A community-developed pipeline called bcbio-nextgen was used to automate the upstream bioinformatic analysis (Guimera, 2011).

#### 3.9.2 Identification and Functional Annotation of Differentially Expressed Genes

The R package bcbioRNASeq can take the bcbio-nextgen output as input and generate count matrices, identify DEGs, conduct functional annotations and visualize the results (Steinbaugh et al., 2018). DEGs were identified for each group compared with the control group using DESeq2, (Love et al., 2014) with a fold-change threshold of 1 and *P* values cutoff of 0.05. Functional annotation of DEGs was performed using the clusterProfiler R package to identify associated pathways in AKI (Yu et al., 2012). We further explored the mechanism of AKI and whether PSCZ can reverse the unfavorable changes in transcriptional level.

### 3.10 Characterization

T Fourier transform infrared spectra (FT-IR) were measured on a Shimadzu FTIR 8400S spectrometer. Scanning electron microscopy (SEM) images were captured on a Hitachi FE-SEM S-4800 instrument with an acceleration voltage of 3 kV. The samples were prepared by depositing sample dispersion onto a freshly cleaved silicon wafers surface. Powder X-ray diffraction (PXRD) patterns were collected on a PANalytical B.V. Empyrean powder diffractometer, in which data were collected from 5° to 45° at a scan rate of 15°/min.

## Data Availability

The datasets presented in this study can be found in online repositories. RNA-seq sequencing results can be found in the NODE database using accession number OEP000285.
