# Supplementary material for: Treatment of Acute Kidney Injury Using a Dual Enzyme Embedded Zeolitic Imidazolate Frameworks Cascade That Catalyzes In Vivo Reactive Oxygen Species Scavenging
Source: Front Bioeng Biotechnol. 2022 Jan 4;9:800428. doi: 10.3389/fbioe.2021.800428 (PMC8764232; doi:10.3389/fbioe.2021.800428)
Supplement: Supplementary file 1 [file DataSheet1.PDF]

Support information

Treatment of acute kidney injury using a dual enzyme embedded Zeolitic imidazolate frameworks cascade that catalyzes *in vivo* reactive oxygen species scavenging

Xinyue Hou[1,2 #], Jianxiang Shi[3#], Jie Zhang[1], Zhigang Wang[1], Sen Zhang[2], Ruifeng Li[2], Wei Jiang[2], Tingting Huang[3], Jiancheng Guo[2,3, *], Wenjun Shang[1,*]*

[1] *Department of Kidney Transplantation, The First Affiliated Hospital of Zhengzhou University, Academy of Medical Sciences, Zhengzhou University, Henan 450052, China.*

[2] *Department of Molecular Pathology, Application Center for Precision Medicine, The Second Affiliated Hospital of Zhengzhou University, Academy of Medical Sciences, Zhengzhou University, Henan 450052, China.*

[3] *College of Chemistry, Jilin University.*

#: These authors contributed equally to this work.

*** Corresponding authors:**

Wenjun Shang

Email: fccshangwj@zzu.edu.cn

Jiancheng Guo

Email: gjc@zzu.edu.cn

Tingting Huang

Email: huangtt18@mails.jlu.edu.cn

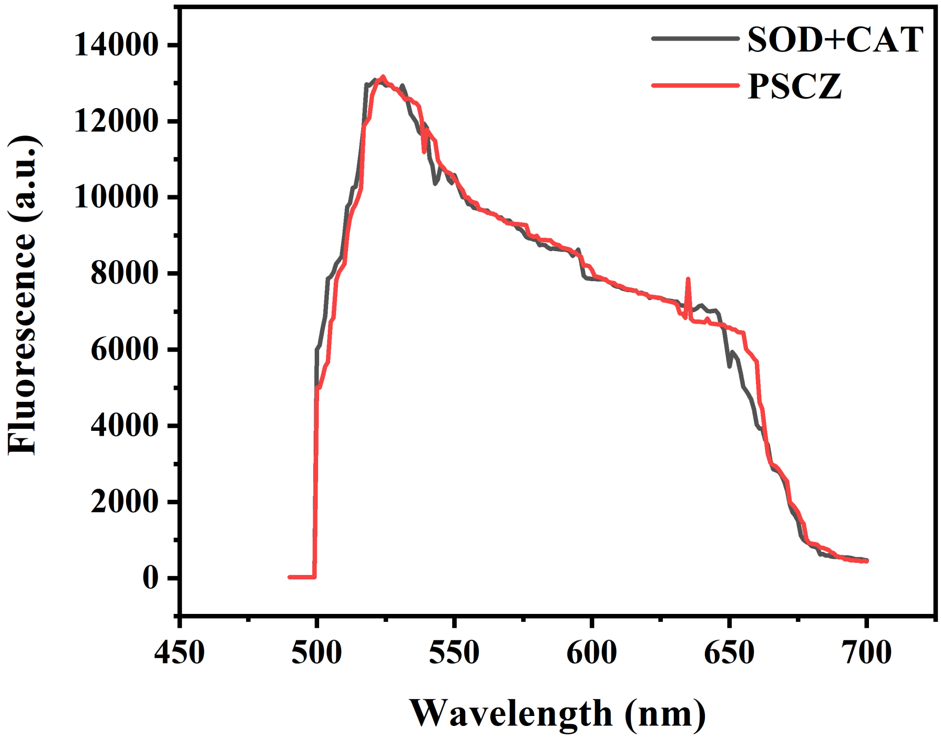

Figure S1 Fluorescence spectra of SOD + CAT and PSCZ. SOD and CAT were labeled with FITC and Rhodamine 6G, respectively. The excitation wavelength used was 450 nm.

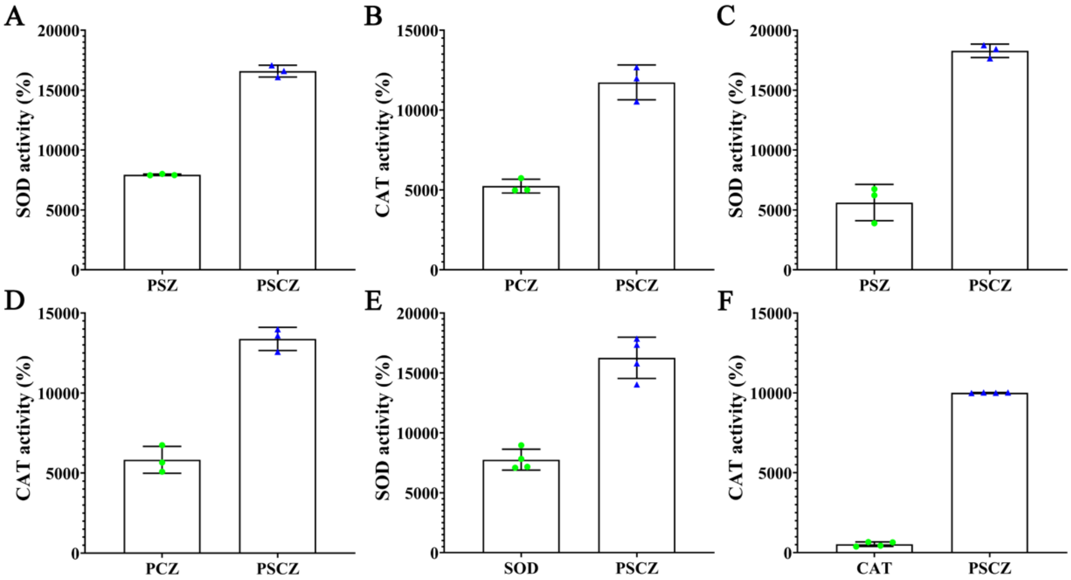

Figure S2 Enzyme activity of PSCZ at different temperatures.

A) SOD activity of SOD and PSCZ at 40°C; B) CAT activity of free CAT and PSCZ at 40°C; C) SOD activity of SOD and PSCZ at 60°C; D) CAT activity of free CAT and PSCZ at 60°C; E) SOD activity of free CAT and PSCZ at 80°C; F) CAT activity of free CAT and PSCZ at 80°C

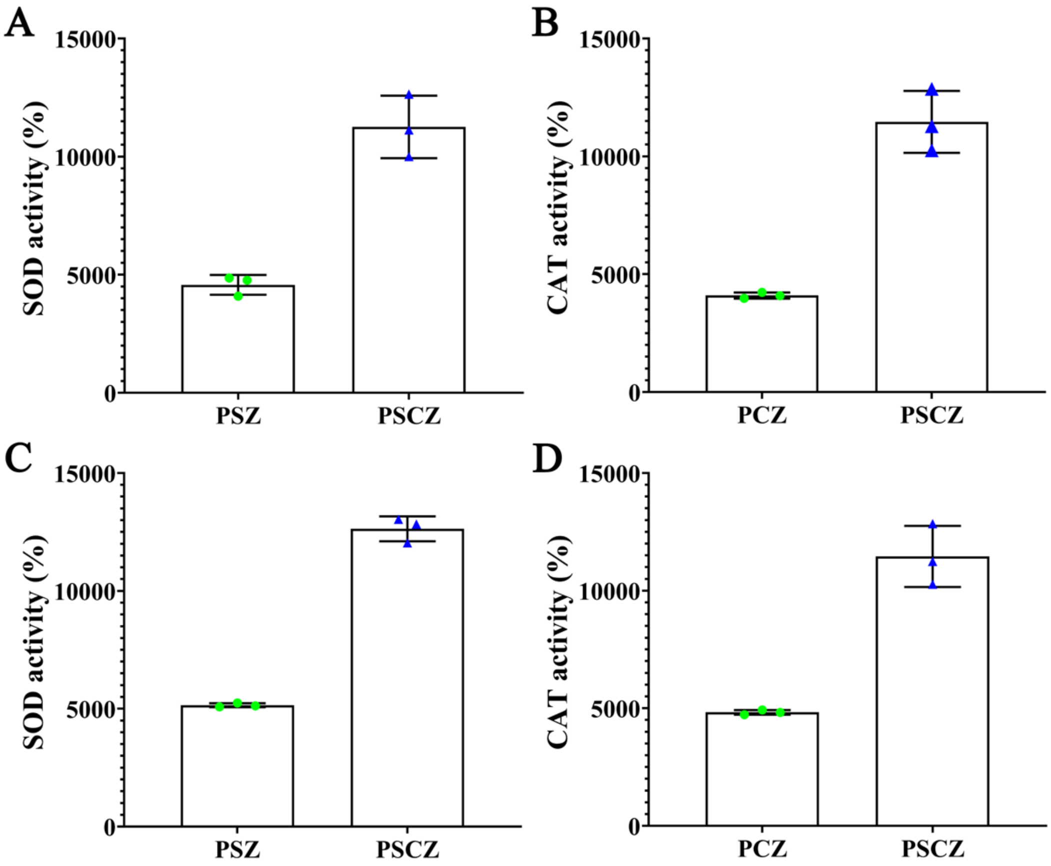

Figure S3 PSCZ enzyme activity under different pH values.

A) SOD activity of SOD and PSCZ at pH 5; B) CAT activity of SOD and PSCZ at pH 5; C) SOD activity of SOD and PSCZ at pH 7.4; D) CAT activity of SOD and PSCZ at pH 7.4

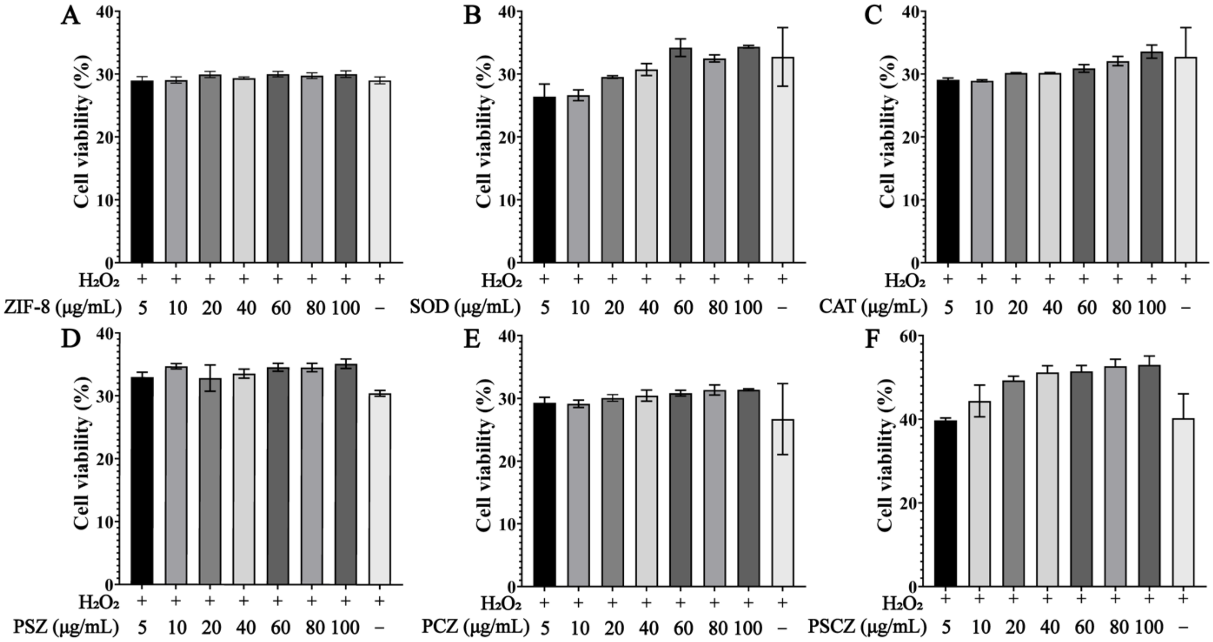

Figure S4. In vitro cell viabilities of HEK293 cells under different treatment conditions.

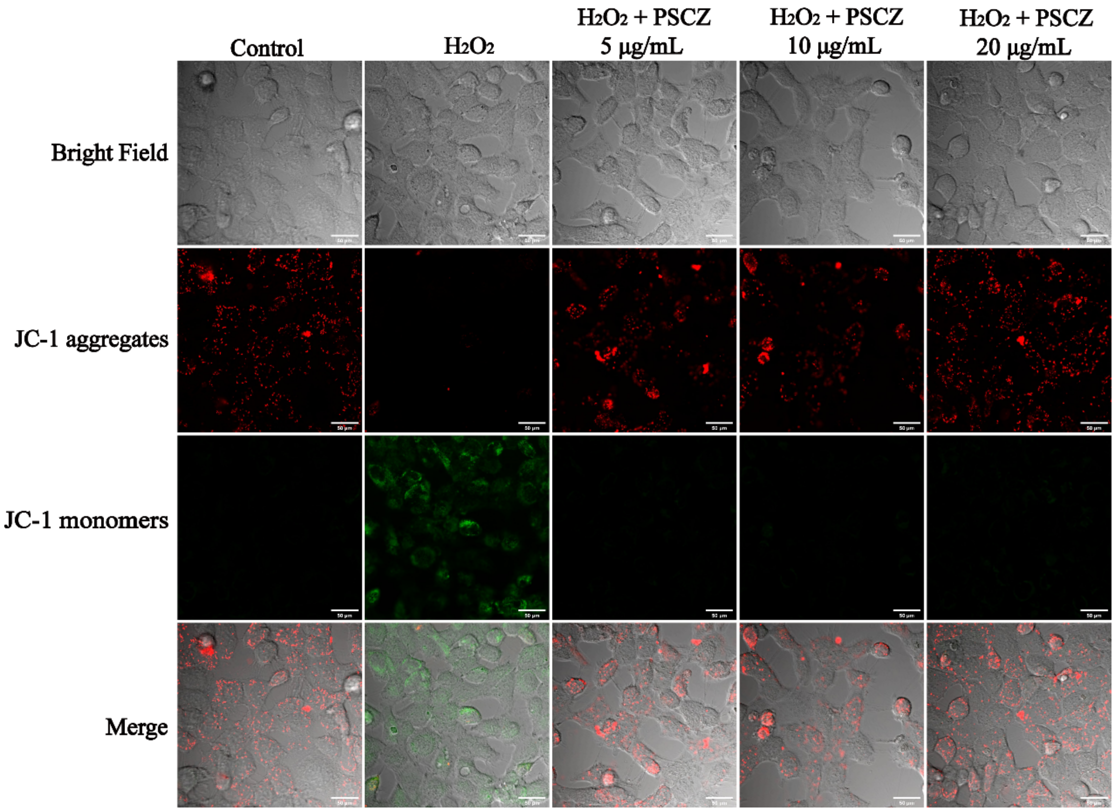

Figure S5 ***In vitro*** **anti-apoptotic activity of MPEG$_{2000}$-SOD@CAT@ZIF-8.** Mitochondrial Δψm values in the indicated treatment groups were assessed via confocal microscopy, scale bar = 50 μm.

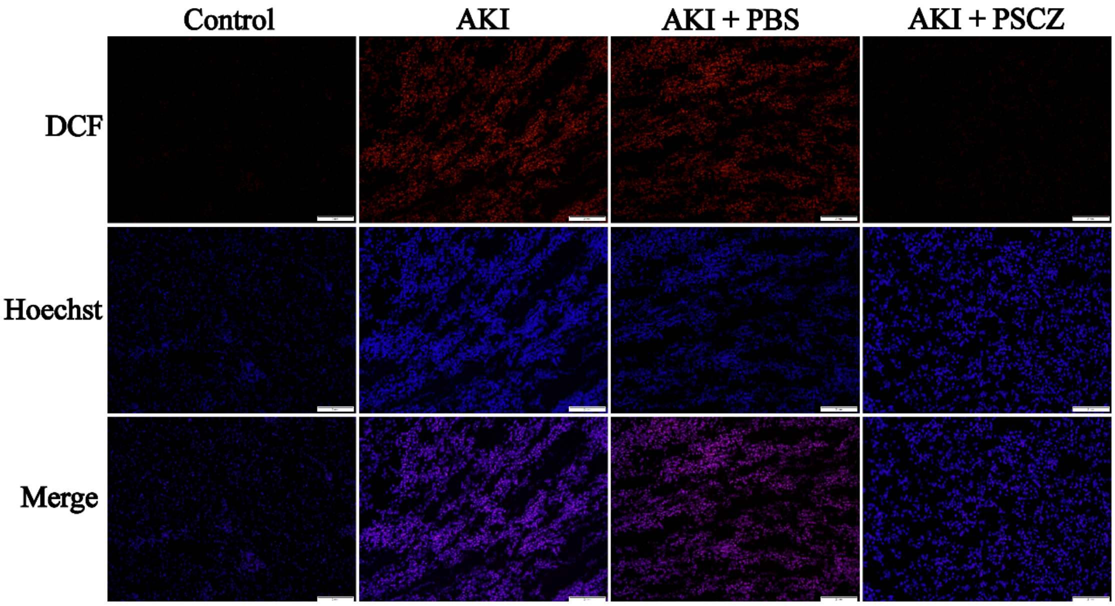

Figure S6 Assessment of MPEG$_{2000}$-SOD@CAT@ZIF-8 therapeutic efficiency in AKI model mice. Renal tissue samples from mice in the indicated treatment groups were assessed via DCFH-DA and Hoechst staining. Scale bar: 50 μm.

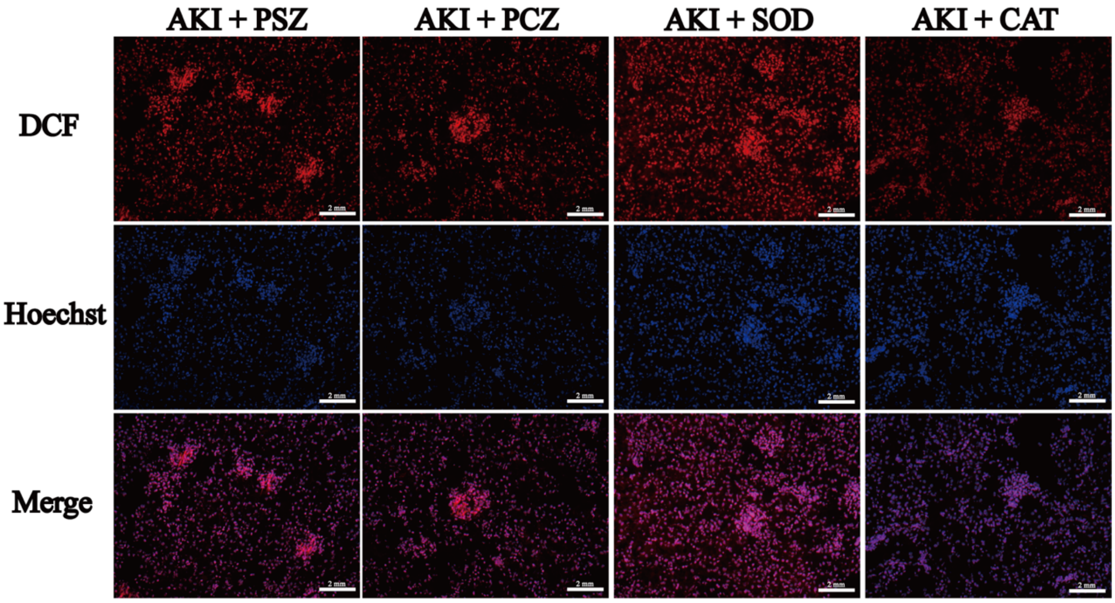

Figure S7 Assessment of PSZ, PCZ, free SOD and free CAT therapeutic efficiency in AKI model mice. Renal tissue samples from mice in the indicated treatment groups were assessed via DCFH-DA and Hoechst staining. Scale bar: 50 μm.

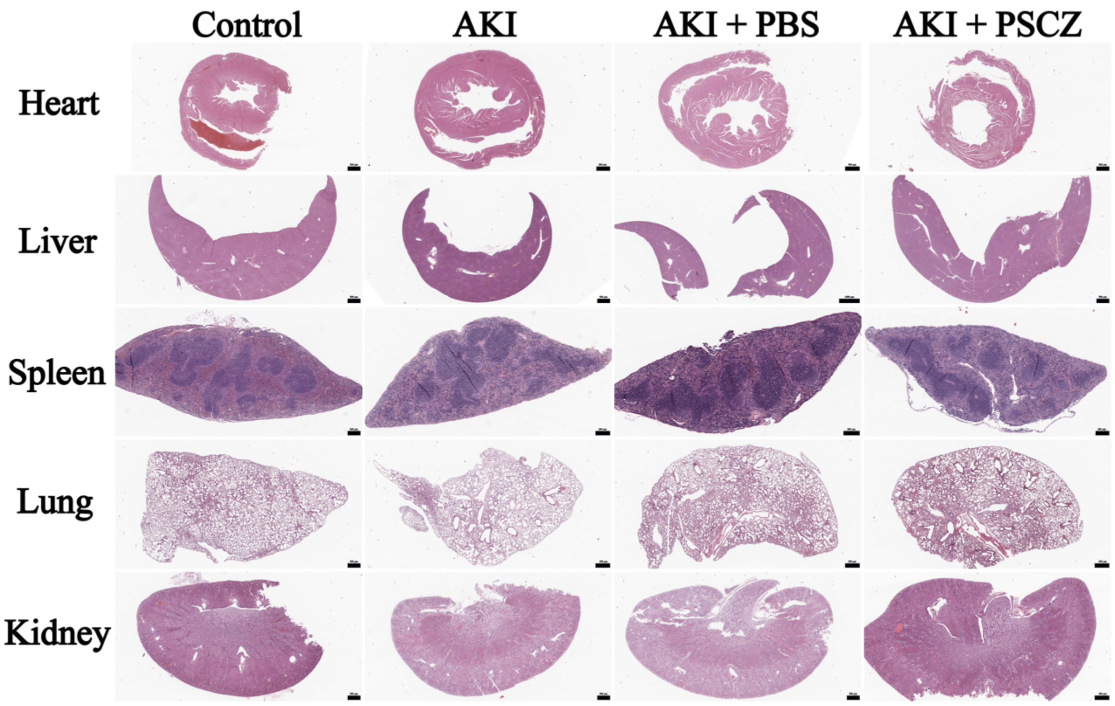

Figure S8 Evaluation of in vivo toxicity of PSCZ to major organs (heart, liver, spleen, and lung) at 24 h after intravenous administration

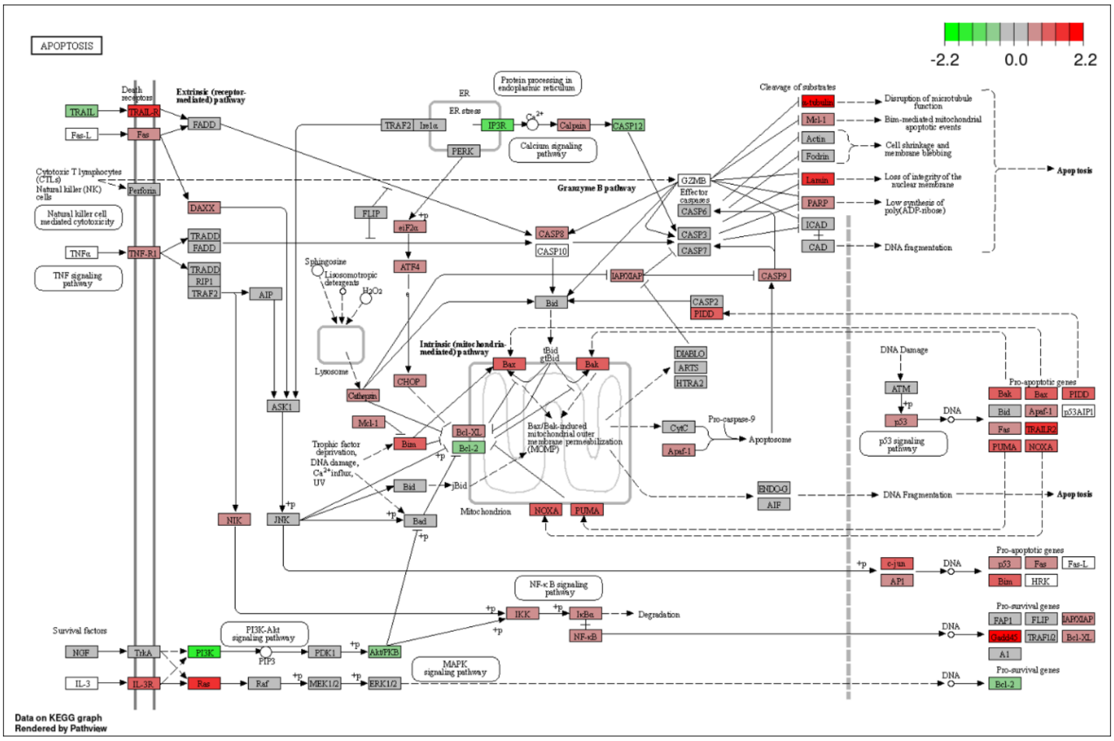

Figure S9 Apoptosis related pathways in the CP-AKI group

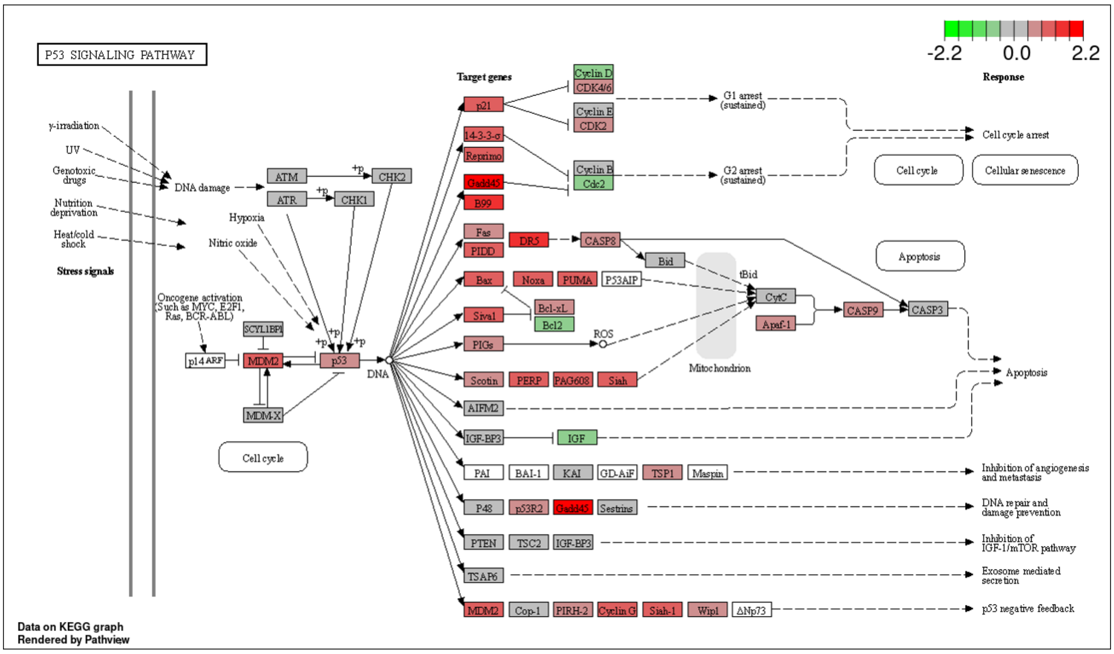

Figure S10 p53 signaling pathway in CP-AKI group
